# Length Scale Matters: Real-Time Elastography versus Nanomechanical Profiling by Atomic Force Microscopy for the Diagnosis of Breast Lesions

**DOI:** 10.1155/2018/3840597

**Published:** 2018-10-16

**Authors:** Rosanna Zanetti-Dällenbach, Marija Plodinec, Philipp Oertle, Katharina Redling, Ellen C. Obermann, Roderick Y. H. Lim, Cora-Ann Schoenenberger

**Affiliations:** ^1^Gynecology and Gynecologic Oncology, Claraspital, 4016 Basel, Switzerland; ^2^Biozentrum and Swiss Nanoscience Institute, University of Basel, Switzerland; ^3^Gynecology and Obstetrics, University Hospital Basel, 4031 Basel, Switzerland; ^4^Faculty of Medicine, University of Basel, 4031 Basel, Switzerland; ^5^Department of Chemistry, University of Basel, Switzerland

## Abstract

Real-time elastography (RTE) is a noninvasive imaging modality where tumor-associated changes in tissue architecture are recognized as increased stiffness of the lesion compared to surrounding normal tissue. In contrast to this macroscopic appraisal, quantifying stiffness properties at the subcellular level by atomic force microscopy (AFM) reveals aggressive cancer cells to be soft. We compared RTE and AFM profiling of the same breast lesion to explore the diagnostic potential of tissue elasticity at different length scales. Patients were recruited from women who were scheduled for a biopsy in the outpatient breast clinic of the University Hospital Basel, Switzerland. RTE was performed as part of a standard breast work-up. Individual elastograms were characterized based on the Tsukuba elasticity score. Additionally, lesion elasticity was semiquantitatively assessed by the strain ratio. Core biopsies were obtained for histologic diagnosis and nanomechanical profiling by AFM under near-physiological conditions. Bulk stiffness evaluation by RTE does not always allow for a clear distinction between benign and malignant lesions and may result in the false assessment of breast lesions. AFM on the other hand enables quantitative stiffness measurements at higher spatial, i.e., subcellular, and force resolution. Consequently, lesions that were false positive or false negative by RTE were correctly identified by their nanomechanical AFM profiles as confirmed by histological diagnosis. Nanomechanical measurements can be used as unique markers of benign and cancerous breast lesions by providing relevant information at the molecular level. This is of particular significance considering the heterogeneity of tumors and may improve diagnostic accuracy compared to RTE.

## 1. Introduction

Breast cancer is the most frequently diagnosed cancer in women. Manifestation of suspicious breast lesions leads to a comprehensive work-up including clinical examination, breast ultrasound, mammography, and, if indicated, magnetic resonance imaging.

Breast ultrasonography (US) is a critical diagnostic tool to characterize breast lesions. On the basis of sonomorphologic characteristics, breast lesions are classified according to the Breast Imaging Reporting and Data System (BI-RADS) (ACR BI-RADS Atlas American College of Radiology 2003) in the following categories: BI-RADS 2 benign, BI-RADS 3 probably benign, BI-RADS 4 suspicious of malignancy, BI-RADS 5 highly suggestive of malignancy, and BI-RADS 6 biopsy-proven malignancy. A sonographic examination that reveals unsuspicious breast tissue is classified as BI-RADS 1.

Despite benign criteria, BI-RADS 3 lesions have been shown to have a malignancy rate of 0.2-11.4% [1–3]. Moreover, in 506 breast lesions classified as BI-RADS 3, biopsy revealed 2.6% to be false negative [3]. Therefore, additional parameters to more accurately predict malignancy are needed.

The difference in mechanical properties between normal and pathologic breast tissue has long been recognized [4]. It provides the basis of manual palpation as well as several noninvasive macroscopic breast imaging techniques where quantitative stiffness contrasts are recorded [5]. For example, elastography uses the stiffness differences of a mass compared to the healthy surrounding tissue for further characterization of the breast lesion. Real-time elastography (RTE) and shear wave elastography are the two modalities used in clinics as a noninvasive adjunct to breast US [6–9]. In RTE, external cyclic compression by the ultrasound probe leads to tissue displacement where soft areas are more readily displaced than harder areas [10]. Strain distribution, which is inversely related to tissue stiffness, is visualized as a color-coded map that is superimposed on the B-mode image of ultrasound [11]. The interpretation of strain images is carried out using the Tsukuba elasticity score (TS) introduced by Itoh et al. [10]. Additional information on tissue elasticity is provided by the strain ratio (SR) between the breast lesion and adjacent fatty tissue [12, 13]. Although elastography in combination with B-mode US improves specificity, accuracy, and positive predictive value (PPV) [9, 14, 15] it does not unambiguously distinguish between benign and malignant lesions.

Besides these macroscopic methods that visualize the mechanical response of the tissue within a breast, other microscopic* ex vivo* techniques are starting to emerge. They employ portable indentation devices that apply either uniaxial cyclic compression or punch indentation to measure mechanical response of a breast biopsy [16, 17]. Recently, a needle-based modulus-sensing probe that was used for modulus measurements of explanted tissue samples has been introduced [18]. These techniques typically do not possess sufficient resolution to detect and evaluate the heterogeneous behavior of malignant breast tumors which would be the crucial improvement to current diagnostic accuracy.

Moreover, efforts to understand cancer biomechanics have been largely polarized between tissue-level (macroscopic) [19] and single-cell experimentation [20]. Macroscopic methods such as RTE show that malignancy is associated with increased stiffness whereas single-cell analysis show that cancer aggressiveness is associated with a softening of cancer cells. This controversy has been bridged by atomic force microscopy (AFM)-type nanomechanical testing, which quantifies local stiffness properties across an entire biopsy at the molecular level [21]. Besides reflecting tumor heterogeneity, nanomechanical AFM profiles can also provide diagnostic information.

In this study, we compare tissue elasticity of different breast lesions at the macroscopic scale measured by RTE* in situ* to the nanomechanical profiles of the corresponding biopsy specimens recorded by AFM.

## 2. Methods

### 2.1. Patient Recruitment

The study was approved by the Ethics Committee of Basel (ref. no. EK:157/08) and conducted in accordance with ethical guidelines. All participating patients provided informed consent. Patients for comparative elasticity analysis were recruited between 2009 and 2012 in the outpatient breast clinic of the Women's Hospital of the University Hospital Basel, Switzerland. Recruitment was carried out among women who were scheduled for an ultrasound-guided core biopsy based on a suspicious solid breast lesion. For this study, we have evaluated 31 patients by AFM and elastography.

### 2.2. Breast Lesion Elasticity at Different Length Scales

**Figure 1:**
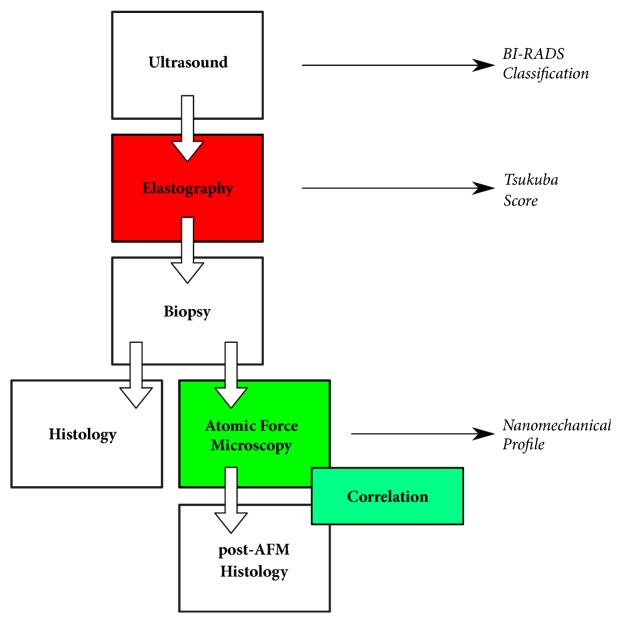
Flow chart of study comparing real-time elastography and AFM nanomechanical testing in the evaluation of breast lesion elasticity from individual patients.

The study flow chart is outlined in Figure 1. After participants received full information about the study and provided a written consent, a breast ultrasound examination was performed using an EUB-7500 Hitachi ultrasound system equipped with a EUP-L74M linear transducer (50mm wide). This transducer allows for conventional B-mode sonography and RTE recordings at the macroscopic scale.

### 2.3. Real-Time Elastography

A routine breast work-up by an experienced physician included an ultrasound examination followed by RTE. For ultrasound, the lesion was scanned in B-mode and the dimensions of the lesion were recorded in three orthogonal planes. Each lesion was categorized from 3 to 5 according to BI-RADS-US assessment categories (ACR BI-RADS Atlas American College of Radiology 2003). Subsequently, RTE was performed. On average, 3-5 elastograms per lesion were recorded under similar conditions. For each measurement, the transducer was placed perpendicular to the skin just above the lesion. The region of interest was selected to include the lesion surrounded by normal breast tissue as well as the subcutaneous tissue and the superficial part of the pectoralis muscle avoiding the rib. The lateral margins were given by the width of the probe (EUP-L74M, 50 mm). The manual freehand cyclic compression/decompression technique [10] was applied with an optimal pressure between 3 and 4 according to the pressure amplitude displayed by the Hitachi software. The tissue deformation was represented by a color-coded elasticity image calculated by the Hitachi software.

On a split-screen, both the US B-mode image, which allows identifying the breast lesion and the normal breast tissue, and an overlay of the color-coded elastogram on the B-mode image are displayed.

### 2.4. Elasticity Assessment

Individual elastograms were characterized based on the principles of the Tsukuba elasticity score developed by Itoh et al. [10]

Lesion elasticity was semiquantitatively assessed by comparing the compression response of the lesion to that of neighboring fatty tissue, which results in a fat to lesion or strain ratio (SR) [12, 13]. To determine the SR, a circular region of interest (ROI A) was selected on a static elastogram, which included as much of the lesion as possible. A second circular region of interest (ROI B) was chosen in the adjacent fatty tissue at a depth similar to that of the lesion. SRs were calculated by the US system's built-in software. Based on previously published data, a cut-off SR value of 2.5 was applied to discriminate between benign and malignant lesions [9].

### 2.5. Ultrasound-Guided Core Biopsy and Pathohistology

For histopathological diagnosis and indentation-type AFM, four to five core biopsy samples were routinely taken from each lesion. All core biopsies (14-gauge needle, Magnum® Core high speed, Bard Medica, Karlsruhe, Germany) were obtained under sonographic guidance. Immediately after removal, one biopsy sample was placed into ice-cold sterile Ringer solution containing 1 tablet of protease inhibitors (Complete, EDTA free, Roche, Switzerland) per 50 ml of Ringer solution (henceforth known as “Ringer Complete”) and processed for nanomechanical AFM profiling with minimal delay. The remaining biopsies were fixed in formaldehyde and processed by standard procedures for examination by an experienced pathologist. Directly after AFM analysis, this sample was also processed for histopathology. All lesions were classified according to the WHO classification [22].

### 2.6. Indentation-Type AFM Measurements

All preparative steps were performed as described previously ([21]; US Patent 8756711B2). Prior to AFM measurement, each specimen was immobilized on a 35 mm plastic cell culture dish (Culture Dish 40, TPP, Switzerland) with a thin layer of two-component fast drying epoxy glue (Devcon). All sample preparation steps were performed in liquid, and mechanical manipulations were kept minimal at all times. Nanomechanical measurements were carried out with a NanoWizard I atomic force microscope (JPK Instruments). To compensate for large surface corrugations on such native biopsies, we developed and implemented customized homebuilt hardware and software algorithms for automated leveling, which enabled uninterrupted AFM operation during data acquisition (US patent US9244095B2). A built-in top-down microscope was used to visually position the AFM cantilever with respect to the specimen. AFM probes that are able to detect local stiffness heterogeneities arising from specific cellular and matrix characteristics were used as described previously [21, 23–25]. In particular, four-sided pyramidal tips DNP-S10 D with a nominal stiffness of 0.06 N/m (Bruker, USA) were used. The exact spring constant k of the cantilever was determined before each experiment using the thermal tune method [26] and the deflection sensitivity was determined in fluid using plastic substrates as an infinitely stiff reference material. Measurements were performed in close to physiological conditions by recording up to 30 different 20-50 *μ*m force maps. Force-volume maps were recorded over 24 × 24 point grids up to 72 × 72 point grids for high spatial resolution. Individual force curves were sampled at 2048 Hz with Z piezo-displacements between 5 and 10 *μ*m, collected at an indentation velocity of 16 *μ*m/s. The applied loading force was set constant to 1.8 nN [27].

### 2.7. Data Analysis

Force curves were analyzed using custom made NuoAnalyzer® (Biozentrum, University of Basel) software according to the modified Oliver and Pharr method [21, 28]. The stiffness values (Pa = N/m^2^) were calculated from force curves and spatially plotted to yield color-coded stiffness maps. 3D overlay maps were created using Gwyddion. Stiffness maps depicting distinct tissue regions were correlated with H&E stained histological sections of the corresponding specimen to ensure that biopsy-wide histological and AFM findings can be compared. The histopathological diagnosis was used as the gold standard for comparison with RTE and AFM data.

### 2.8. Statistics of AFM Measurements

All individual stiffness values for a specimen were added up in NuoAnalyzer® to obtain the distribution of stiffness values (henceforth defined as biopsy-wide histogram). The bin width was set to 50 Pa for all specimens for individual and biopsy-wide histograms, and counts were normalized in the interval 0 to 1. Predominantly, biopsy-wide histograms followed log-normal distributions where the maximum of the peak represents a mean stiffness value and the width of the distribution corresponds to its standard deviation. For data fitting, a multipeak fit was applied for all stiffness distributions where the distribution peaks were located using the peak analysis in the “Fitting Tool” (NuoAnalyzer®). The statistical significance of differences in mean values was assessed with the paired Student's t-test in OriginPro 2015G. Statistical significance was set at P ≤ 0.05.

## 3. Results

**Figure 2:**
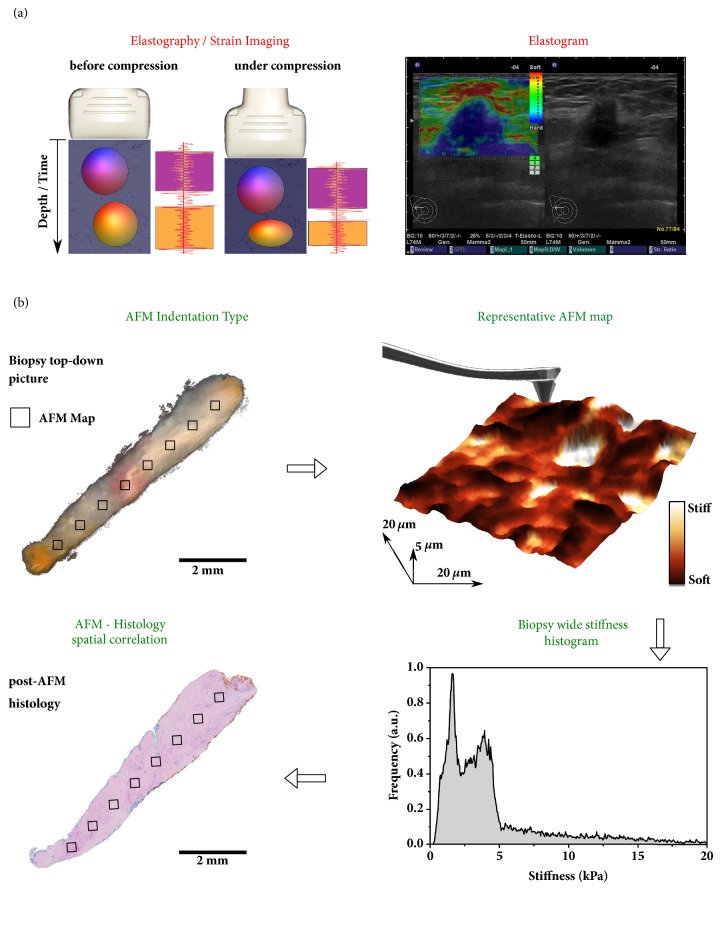
**The spatial and mechanical sensitivity of real-time elastography and atomic force microscopy**.** (a)** (Left) Real-time elastography provides a “strain image” or an elastogram based on the deformation of a breast lesion related to a compression force cyclically applied to the breast by a hand-held probe. (Right) Real-time elastography read-out from the Hitachi instrument used in this study. In the elastogram, tissue stiffness is color-coded and superimposed on the B-mode image. The example shows an invasive breast carcinoma (of 14.7mm diameter).** (b)** Top view image of an ultrasound-guided biopsy from a suspicious breast lesion immersed in a Ringer solution. Up to 20 stiffness maps (20 × 20 *μ*m^2^; represented by black squares), each consisting of 1024 to 4096 indentation measurements, are recorded in a grid across the entire specimen. Subsequent analysis of the data provides a biopsy-wide stiffness distribution and individual areas can be visualized plotting color-coded stiffness maps. The choice of dimensions for both the AFM tip and the cantilever (drawn on the stiffness map) is critical for obtaining high-resolution topography and the nanomechanical properties. Post-AFM histology (bottom left) was used for spatial correlation of AFM maps and confirmation of AFM-based diagnostics.

In RTE (Figure 2(a)), manually applied cyclic compression by a probe results in differential breast tissue displacement in that stiffer regions are less prone to displacement than softer regions [10]. Stiffness of the scanned tissue, which is inversely correlated to the recorded strain images, is visualized as a color-coded elastogram that is superimposed on the B-mode image of conventional US. For example, the elastogram in Figure 2(a) reveals a stiff area (blue) with dimensions that are slightly larger than those in the B-mode image. The high stiffness as well as the ultrasound features in the B-mode image are indicative of an invasive breast cancer.

In contrast to RTE, which records the bulk stiffness of lesions* in situ*, indentation-type AFM has evolved as a procedure to measure nanoscale stiffness of breast lesion biopsies at the cellular level [21]. As outlined in Figure 2(b), individual 20 × 20 *μ*m AFM stiffness maps are collected across the entire surface of fresh biopsy specimens. The frequency of specific stiffness values is represented by a biopsy-wide histogram, revealing a nanomechanical signature of each lesion. Post-AFM H&E stained histological sections were used to associate biopsy-wide stiffness profiles with tissue composition (Figure 2(b), bottom left).

**Figure 3:**
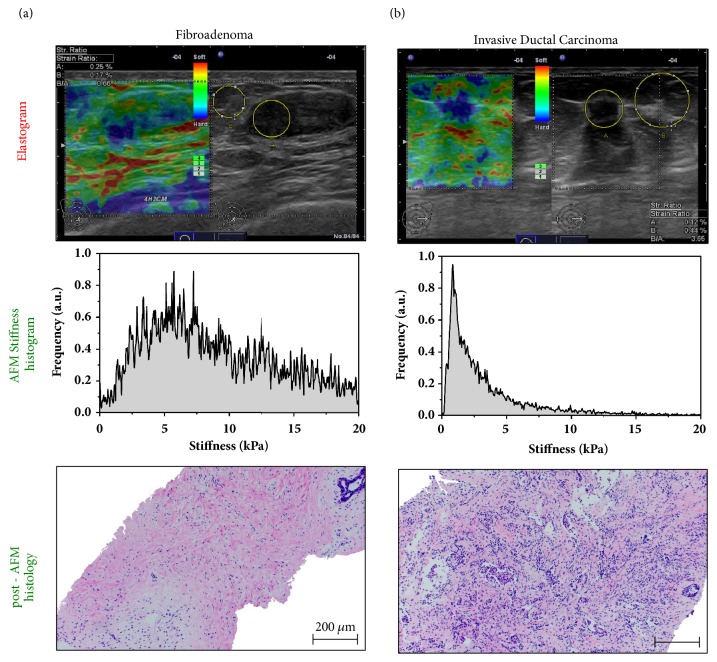
**Mechanical response of benign and cancerous breast lesions assessed by elastography and AFM nanomechanical testing**.** (a)** (Top) Fibroadenoma: in B-mode image oval circumscribed hypoechogenic lesion of 22.3mm diameter, assessed as BI-RADS 3; elastogram reveals lesion with little strain shown as mosaic pattern of blue and dominantly green assessed as Tsukuba score 2, strain ratio 0.66. (Middle) AFM testing of a biopsy from the lesion in (a) shows a unimodal but broad stiffness distribution indicating both cellular and extracelluar components within the specimen. The histogram peaking at the maximum value of 6 kPa reveals strong stromal response as illustrated by the H&E image recorded post-AFM on the same specimen (bottom). Scale bar, 200 *μ*m.** (b)** (Top) Invasive ductal breast cancer: in B-mode image irregular hypoechogenic lesion of 8.8mm diameter with architectural distortion, assessed as BI-RADS 5, lesion with even strain in the lesion as well as in the surrounding tissue assessed as Tsukuba score 5, strain ratio 3.65. (Middle) The AFM data show heterogeneous stiffness distribution with a soft peak at 0.6 kPa followed by an exponential decay, which is characteristic of malignant tumor. (Bottom) This is consistent with the post-AFM histopathology, revealing an invasive ductal breast carcinoma with infiltrating nests of cancer cells that have evoked a desmoplastic tissue response. Scale bar, 200 *μ*m.

To compare tissue elasticity at different length scales, RTE and AFM testing was performed on the same breast lesion (Figure 3). The B-mode image in Figure 3(a) (right panel) revealed an oval circumscribed hypoechogenic lesion which was classified as BI-RADS 3 (probably benign). As shown by the corresponding elastogram (left panel), lesion strain was similar to that of the surrounding healthy breast tissue. Based on the mosaic pattern of green and blue the lesion qualifies as TS 2 (benign). The lesion to fat strain ration (SR) of 0.66 was consistent with a benign breast lesion [9]. AFM testing of a fresh biopsy from this lesion revealed a detailed picture of stiffness at the cellular level (middle panel). The stiffness distribution across the biopsy was broad but unimodal with a peak value at 6 kPa. This stiffness profile is typical for a benign lesion [21]. Stiffness values around 1-2 kPa were representative of cellular components whereas the large fraction of higher stiffness values reflected an increase of stromal matrix within the specimen. The strong stromal response in this specimen was confirmed by post-AFM histology (bottom panel). Consistent with RTE and AFM, histopathology demonstrated a fibroadenoma.

In the case of an invasive ductal carcinoma (Figure 3(b)), B-mode image demonstrated a speculated lesion that interrupted the normal breast architecture. Based on the sonomorphology, the lesion was classified as BI-RADS 5 (highly suggestive of malignancy). As revealed by the dark blue color in the elastogram, the lesion exhibited only little strain which was homogenously distributed across the entire lesion and an SR of 3.65. Typically, the area representing the relatively hard tissue in the elastogram was larger than the hypoechogenic area detected in the B-mode image, reflecting the desmoplastic reaction. These elastographic features correspond to a TS5 (malignant) lesion. Consistently, the histopathologic examination of biopsy specimens diagnosed an invasive ductal carcinoma.

One biopsy specimen was examined by AFM testing (middle panel). The stiffness distribution across the specimen revealed a prominent soft peak at 0.6 kPa indicative of malignancy [21] with an exponential decay of stiffness values. The post-AFM histopathology (bottom panel) confirmed the prevalence of cancer cells surrounded by little stroma.

**Figure 4:**
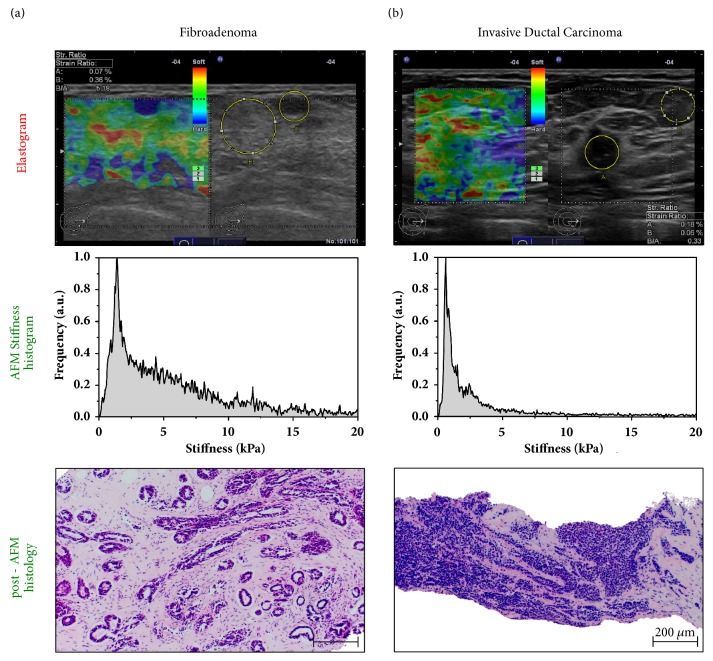
**Stiffness sensitivity and spatial resolution of nanomechanical testing improves diagnostic accuracy**.** (a)** (Top) Fibroadenoma: in B-mode image, oval circumscribed hypoechogenic lesion of 5.1mm diameter with a BI-RADS score of 3. The even strain in the lesion and in the surrounding tissue at a strain ratio of 5.18 led to a Tsukuba score of 5 (malignant). Histology revealed this false positive. (Middle) In contrast to elastography, the high spatial and force sensitivity of the AFM allowed for a clear distinction of the cellular and extracellular matrix (ECM) components. The biopsy-wide histogram reveals a broad stiffness distribution up to 20 kPa arising from the stiff ECM components with a prominent peak around 2 kPa that is correlated well with the high glandular content in the lesion as corroborated by the post-AFM histology image (bottom). Scale bar, 200 *μ*m.** (b)** (Top) Invasive ductal breast cancer: B-mode image shows an irregular hypoechogenic lesion of 21 mm diameter with architectural distortion, assigned a BI-RADS score of 5. RTE reveals a lesion of little strain reflected by a mosaic pattern of blue and dominantly green in the elastogram assigned a Tsukuba score of 2 (false negative score) (strain ratio 0.33). (Middle) The biopsy-wide histogram of AFM stiffness recordings reveals a bimodal stiffness distribution typical for invasive breast cancer. The prominent and narrow stiffness peak dominating at 0.6 kPa is a feature of cancer cells. (Bottom) Post-AFM histology shows large areas with densely packed cancer cells and low stromal content being present in the biopsy, which might explain the strain ratio and false Tsukuba score. Scale bar, 200 *μ*m.

In some cases, evaluation of stiffness at the macroscopic level may lead to a false assessment of breast lesions. Examples of a false positive and a false negative RTE are shown in Figure 4.

The elastogram in panel (a) reveals an even strain throughout the lesion with an SR of 5.18. Based on RTE, this lesion was classified as TS5 (malignant). However, testing stiffness across the biopsy specimen at higher spatial and force resolution by AFM revealed the broad stiffness distribution. The prominent peak around 2 kPa is indicative of a high amount of stromal, i.e., fibroblast cells, whereas the extracellular matrix is represented by values up to 20 kPa. Consistently, post-AFM histology confirmed the diagnosis of a fibroadenoma with a significant number of fibroblasts.

In contrast, the elastogram in Figure 4(b) had an SR of 0.33 and was scored as TS 2 which suggests a benign lesion. AFM testing revealed a dominating, narrow stiffness peak at 0.6 kPa which is typical for breast cancer cells [21]. Post-AFM histology showed an invasive ductal breast cancer with large areas of densely packed cancer cells and low stromal content. This finding is inconsistent with the mosaic pattern of blue and green in the elastogram (top panel) that resulted in TS 2.

**Figure 5:**
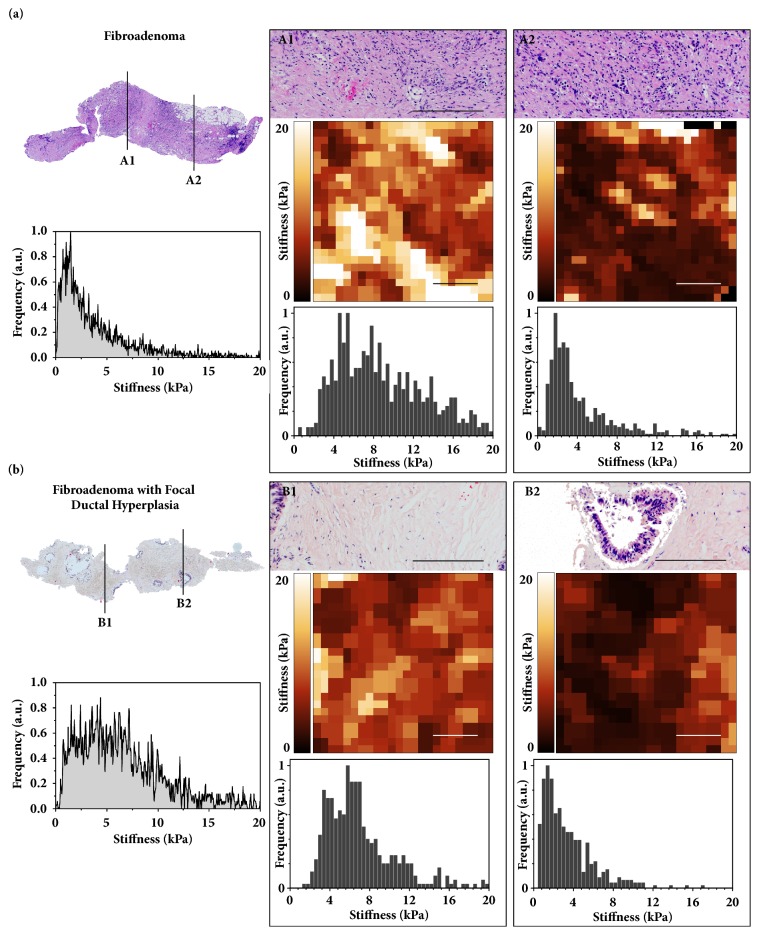
**The nanomechanical signature of benign breast lesions**.** (a)** (left, top) Post-AFM histology of the biopsy revealing a fibroadenoma with reference to the areas A1/A2 mapped in detail. (Left, bottom) Biopsy-wide stiffness distribution shows broad unimodal distribution with a peak value around 2.5 kPa. Local histology analysis (A1/A2 top; scale bar, 200 *μ*m) of mapped regions revealed high abundance of fibroblasts and collagen fibers that could be distinguished in more detail in representative high-resolution AFM stiffness maps (A1/A2 middle; scale bar 6 *μ*m) and by the corresponding stiffness distributions (A1/A2 bottom).** (b)** (Left, top) Post-AFM histological overview of the biopsy revealing a fibroadenoma with focal ductal hyperplasia with reference to the areas B1/B2 mapped in detail. (Left, bottom) Biopsy-wide stiffness distribution shows broad bimodal stiffness distribution with the peak values around 1.5 kPa and 5 kPa. (B1/B2 top) Local histological analysis shows mostly fibrotic tissue in B1, and in B2 an area presenting a duct with usual ductal hyperplasia surrounded by the interstitial connective tissue (scale bar, 200 *μ*m). The hyperplasia is seen as a softer peak in corresponding high-resolution AFM stiffness maps (B2 middle, right; scale bar 6 *μ*m) and a corresponding stiffness distribution typical for healthy tissue (B2 bottom, right). In the adjacent areas, the presence of connective tissue typical of a fibroadenoma was demonstrated by the increased stiffness visualized in the color-coded stiffness map (B1 middle, left; scale bar 6 *μ*m) and by the corresponding stiffness values around 5 kPa (B1 bottom, left).

In macroscopic stiffness evaluation by RTE stromal components let the tumor appear stiffer than the surrounding tissue. As shown by the false positive and false negative TS classification in Figure 4, resolution at the macroscopic level does not always allow for a clear distinction between benign and malignant lesions as cellular components are not recognized. In contrast, nanomechanical testing by AFM enables stiffness measurements at the (sub)cellular level.  Figure 5 illustrates the nanomechanical signature of benign breast lesions. In panel (a), the biopsy-wide stiffness distribution of a fibroadenoma was analyzed in more detail by recording AFM stiffness maps at the locations indicated. Consistent with the post-AFM histology that revealed a high amount of collagen fibers in addition to fibroblasts, the stiffness values in A1 were distributed over a wide range up to 20 kPa.

In A2, the increased cellular content is represented by a peak of stiffness values around 2 kPa. A peak of similar stiffness was found in B2 which represents an area with normal ductal epithelium surrounded by connective tissue. Areas with predominantly connective tissue (B1) were stiffer with stiffness values distributed around 5 kPa. The stiffness distribution shown in Figure 5 demonstrates that nanomechanical testing allows distinguishing between stromal and cellular components.

**Figure 6:**
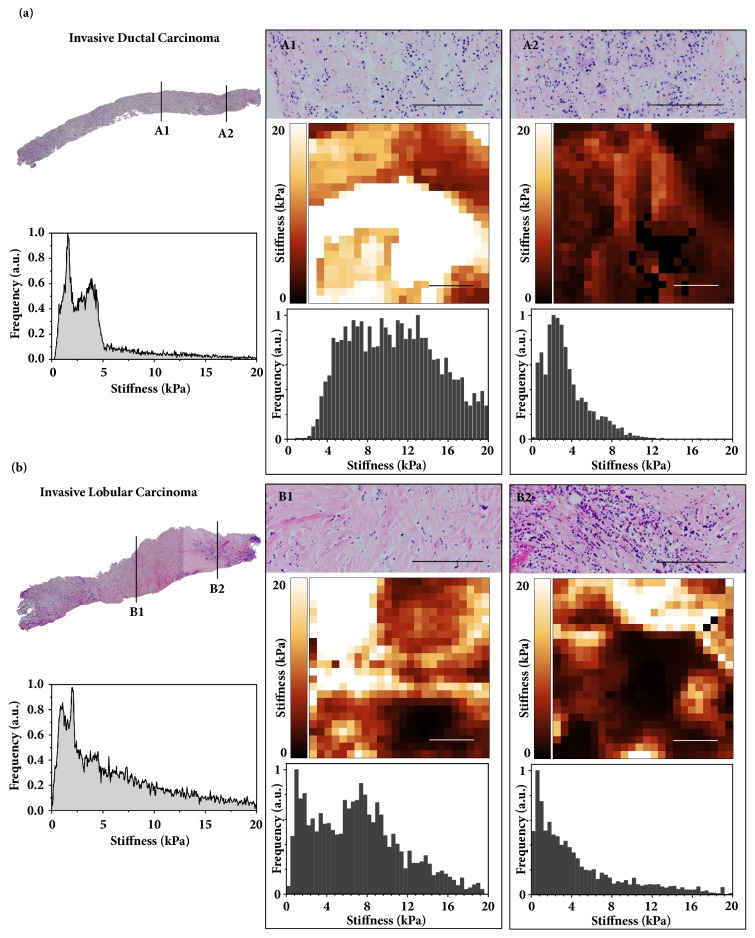
**The nanomechanical signature of prevailing breast cancer types**.** (a)** (Left, top) Post-AFM histological overview of the entire biopsy exhibiting invasive ductal breast carcinoma with reference to the areas A1/A2 mapped in detail on the right (scale bar, 200 *μ*m). (Left, bottom) Biopsy-wide stiffness distribution showing stiffness heterogeneity with a prominent soft peak around 0.6 kPa followed by the second peak at around 3 kPa characteristic of the malignant phenotype. Local histology analysis (A1/A2 top, right) of mapped regions revealed high abundance of cancer cells tending to form glandular structures typical for the invasive ductal carcinoma surrounded by the ECM. This is revealed in more detail in the representative high-resolution stiffness maps (A1/A2 middle; scale bar 6 *μ*m) and by the corresponding bimodal stiffness distributions (A1/A2 bottom).** (b)** (Left, top) Post-AFM histological overview of the lobular carcinoma with reference to the areas B1/B2 mapped in detail (scale bar, 200 *μ*m). (Left, bottom) Biopsy-wide stiffness distribution shows broader heterogeneous stiffness distribution than in the case of ductal carcinoma with values up to 20 kPa and prominent soft peak at 0.6 kPa. Local histological analysis (B1/B2 top, right) revealed areas with less cohesive cellular regions with cells that tend to invade in single file. This is also illustrated by the high-resolution stiffness map (B1/B2 middle; scale bar 6 *μ*m) and corresponding stiffness distribution (B1/B2 bottom).

Correspondingly, the higher resolution of AFM compared to RTE reveals cellular and stromal components in breast cancer (Figure 6). However, compared to benign lesions (Figure 5), the biopsy-wide stiffness distribution of invasive breast cancer is more heterogeneous with a typical prominent soft peak at 0.6 kPa. The invasive lobular carcinoma (Figure 6(b)) showed a broader stiffness heterogeneity than the invasive ductal carcinoma (Figure 6(a)). High-resolution AFM maps revealed in more detail the bimodal stiffness distribution brought about by the high abundance of cancer cells (A2, B2) and the stromal response (A1, B1).

## 4. Discussion

From the early times of mankind, the mechanical stiffness of tumors has helped their detection by manual palpation. At the macroscopic level, tumor-associated changes in tissue architecture are recognized as increased stiffness of the lesion compared to surrounding normal glandular breast tissue. In clinical examination, RTE exploits this difference to describe desmoplasia and other processes that stiffen tissues including ultrastructural changes to the inter- and intralobular stroma that affect the water content of the extracellular matrix and how collagen fibers are crosslinked in breast lesions [29–32]. We and others have shown that RTE complements conventional breast US through diagnostic stiffness information that increases specificity and PPV [8, 9, 14, 15, 33].

However, the accurate distinction of benign versus malignant by RTE is problematic because of ambiguities related to the examiner dependence of RTE, but also because of the features of the lesion (Figure 3; [15, 34]). Although carcinomas are generally hard, soft invasive cancers are seen which do not fulfill the criteria of malignancy defined for RTE and thus result in a false negative result as shown in the RTE of an invasive ductal carcinoma in Figure 4(b). Likewise, as shown in the elastogram of Figure 4(a), a false positive result may be obtained from benign lesions that exhibit the characteristics of a carcinoma. Because atypical macroscopic elasticity in malignant and benign lesions interferes with reliable diagnosis in RTE, more detailed elasticity information is needed for an increased diagnostic accuracy.

In addition, macroscopic elasticity measured by RTE reflects predominantly the stromal component/response of the lesion [30, 35] whereas the higher spatial and force resolution provided by nanoscopic AFM measurements allows for distinguishing between cellular and stromal components [21]. In fact, AFM elasticity profiling reveals that invasive cancer cells exhibit a typical soft phenotype (Figure 3) that is significantly softer than normal breast epithelium, whereas stromal components are relatively hard. Consistent with this notion, our data show that, in contrast to RTE, nanomechanical AFM profiles correctly identified the false negative lesion as malignant and the false positive lesion as benign (Figure 4). Our findings clearly indicate that the increased stiffness sensitivity and spatial resolution offered by AFM improve the reliability of elasticity as a diagnostic marker. In particular, accounting for tumor heterogeneity by the cellular resolution of AFM stiffness measurements and the availability of nanomechanical profiles within hours outweigh the requirement of a breast biopsy.

## 5. Conclusion

RTE is a noninvasive imaging modality that can be easily applied in routine clinical breast work-up. It complements US by further characterizing breast lesions based on their overall elasticity. However, benign lesions that are macroscopically stiffer than usual, such as calcified fibroadenoma, lead to false positive results. Likewise, malignant lesions that are untypically soft, e.g., mucinous carcinomas, appear benign in RTE. In contrast to the bulk stiffness measured by RTE, AFM offers higher spatial resolution and higher sensitivity in assessing stiffness features. Consequently, lesions that were false positive or false negative by RTE were correctly identified by their nanomechanical AFM profiles as confirmed by histological diagnosis. Although a biopsy is required for AFM testing, nanomechanical stiffness profiles can be obtained within a few hours whereas final histological diagnosis is more time consuming.

Nanomechanical measurements can be used as unique markers of benign and cancerous breast lesions by providing relevant information at the molecular level. This is of particular significance considering the heterogeneity of tumors and may contribute to the improvement of diagnostic accuracy compared to RTE. This encourages further development of AFM into a clinical tool for breast diagnostics.

## Data Availability

The data used to support the findings of this study are currently under embargo while the research findings are commercialized. Requests for data 12 months after publication of this article will be considered by the corresponding author.

## Abbreviations

AFM: Atomic force microscopy
BI-RADS: Breast Imaging Reporting and Data System
PPV: Positive predictive value
RTE: Real-time elastography
SR: Strain ratio
TS: Tsukuba elasticity score
US: Ultrasonography.
